# Establishment of canine hemangiosarcoma xenograft models expressing endothelial growth factors, their receptors, and angiogenesis-associated homeobox genes

**DOI:** 10.1186/1471-2407-9-363

**Published:** 2009-10-14

**Authors:** Atsushi Kodama, Hiroki Sakai, Satoko Matsuura, Mami Murakami, Atsuko Murai, Takashi Mori, Kouji Maruo, Tohru Kimura, Toshiaki Masegi, Tokuma Yanai

**Affiliations:** 1 Laboratory of Veterinary Pathology, Department of Veterinary Medicine, Gifu University, Gifu, Japan; 2 Laboratory of Veterinary Clinical Oncology, Department of Veterinary Medicine, Gifu University, Gifu, Japan; 3 Center for Experimental Animals, National Institutes of Natural Sciences, Okazaki, Japan; 4 Research Fellow of the Japan Society for the Promotion of Science, Tokyo, Japan

## Abstract

**Background:**

Human hemangiosarcoma (HSA) tends to have a poor prognosis; its tumorigenesis has not been elucidated, as there is a dearth of HSA clinical specimens and no experimental model for HSA. However, the incidence of spontaneous HSA is relatively high in canines; therefore, canine HSA has been useful in the study of human HSA. Recently, the production of angiogenic growth factors and their receptors in human and canine HSA has been reported. Moreover, the growth-factor environment of HSA is very similar to that of pathophysiological angiogenesis, which some homeobox genes regulate in the transcription of angiogenic molecules. In the present study, we established 6 xenograft canine HSA tumors and detected the expression of growth factors, their receptors, and angiogenic homeobox genes.

**Methods:**

Six primary canine HSAs were xenografted to nude mice subcutaneously and serially transplanted. Subsequently, the expressions of vascular endothelial growth factor (VEGF)-A, basic fibroblast growth factors (bFGF), flt-1 and flk-1 (receptors of VEGF-A), FGFR-1, and angiogenic homeobox genes HoxA9, HoxB3, HoxB7, HoxD3, Pbx1, and Meis1 were investigated in original and xenograft tumors by histopathology, immunostaining, and reverse transcription polymerase chain reaction (RT-PCR), using canine-specific primer sets.

**Results:**

Histopathologically, xenograft tumors comprised a proliferation of neoplastic cells that were varied in shape, from spindle-shaped and polygonal to ovoid; some vascular-like structures and vascular clefts of channels were observed, similar to those in the original tumors. The expression of endothelial markers (CD31 and vWF) was detected in xenograft tumors by immunohistochemistry and RT-PCR. Moreover, the expression of VEGF-A, bFGF, flt-1, flk-1, FGFR-1, HoxA9, HoxB3, HoxB7, HoxD3, Pbx1, and Meis1 was detected in xenograft tumors. Interestingly, expressions of bFGF tended to be higher in 3 of the xenograft HSA tumors than in the other tumors.

**Conclusion:**

We established 6 xenograft canine HSA tumors in nude mice and found that the expressions of angiogenic growth factors and their receptors in xenograft HSAs were similar to those in spontaneous HSA. Furthermore, we detected the expression of angiogenic homeobox genes; therefore, xenograft models may be useful in analyzing malignant growth in HSA.

## Background

Hemangiosarcoma (HSA) is a malignant neoplasm that originates from vascular endothelial cells (ECs). In general, high-grade, aggressive tumors tend to recur locally and metastasize early, despite therapeutic modality [1-4]. However, the mechanisms of tumorigenesis in HSA have been barely elucidated, since these tumors comprise only approximately 2% of all cases of soft-tissue sarcoma in humans. In contrast, spontaneous HSA occurs more frequently in dogs than in any other species [5]; in fact, there are more occasions to gain new insights into canine HSA than into human HSA [6-8]. Knowledge obtained from the study of canine HSA is useful in understanding human HSA. In recent studies into canine and human HSA, expressions of both vascular endothelial growth factor A (VEGF-A) and their receptors, especially flk-1, were evident, and the presence of an autocrine or paracrine mechanism underpinning the growth factors might be associated with the malignant proliferation of canine HSA [9,10]. Considering the similarities shared by human and canine HSAs, a canine HSA model may be useful in the study of human HSA.

Homeobox genes encode homeodomain-containing proteins, which regulate morphogenesis and cell differentiation as a transcription factor in embryonic development. Homeobox genes are highly conserved among various species, and thus they may serve a function similar to that of the corresponding genes in various animals, from simple organisms to vertebrates, including humans [11,12]. Some homeobox genes tightly regulate the behavior of ECs during VEGF or bFGF-induced angiogenesis [13-18]. Regarding growth factor environments, HSA may have a growth mechanism similar to that of pathophysiological angiogenesis, and angiogenesis-associated homeobox genes may play a function in HSA. We hypothesized that angiogenic homeobox genes are also associated with the malignant behavior of HSA, which is rendered possible by the proliferation and invasion of neoplastic ECs.

Several *in vitro *models have been established and used to carry out biological and molecular characterization of canine HSA [19,20]. On the other hand, experimental *in vivo *study models are essential for dynamic analysis of neoplasm. For instance, the pharmaceutical analysis of anticancer drugs with a consideration of drug metabolism is essential because the handling of clinical or fixed histopathological tumor samples is restricted. A transplantable neoplastic model is useful in the dynamic analysis of neoplasm *in vivo*; in human medical research, tumor xenografts have been widely used and have greatly contributed to advancements in the medical sciences [21-23].

In the present study, we attempted to transplant xenografts of canine HSA tumors into athymic nude mice. We were able to establish 6 xenograft tumors derived from 6 spontaneous canine HSAs and subsequently analyze the expression of VEGF-A, basic fibroblast growth factor (bFGF), their receptors, and angiogenesis-associated homeobox genes in established canine HSA xenograft tumors.

## Methods

### Sources of Canine Hemangiosarcoma Tumors

A total of 6 xenograft tumors (Si, Re, Ud, Sy, Sa, and Ju) were established from 6 spontaneous canine HSAs that were collected from the Veterinary Teaching Hospital of Gifu University or from private animal hospitals. Re was obtained during necropsy of a dog with systemic metastasized HSA and the remaining 5 samples were obtained by surgical excision. Information of dogs with original tumors is provided in Table 1.

**Table 1:** An information of original tumors

Xenografted tumor	Breed	Sex	Age	Metastasis	Transplantation tissue
Si	Mix	SF	13 y		Spleen
Re	Golden retriever	CM	10 y	Systemic metastasis	Right auricle
Ud	Papillon	F	11 y		Spleen
Sy	Golden retriever	M	7 y		Left elbow
Sa	Husky	CM	11 y	Liver and inguinal subcutis	Spleen
Ju	Labrador retriever	SF	10 y		Liver

CM: castrated male, SF: spayed female.

After surgical removal or necropsy, these tumors were immediately prepared for histopathology and transplantation. For histopathological examination, these tumors were fixed in 10% neutral buffered formalin and embedded in paraffin, in a routine manner. Tissue sections from the original tumors were stained with hematoxylin and eosin (HE), as is customary. To transplant tumor to mice, tumor tissues were cut into 1-mm^3 ^cubes under sterile conditions.

### Nude Mice and Transplantations of Canine HSA Tissue to Nude Mice

Three-week-old male KSN Slc mice (Japan SLC, Inc., Hamamatsu, Japan) were anesthetized with pentobarbital and subcutaneously xenografted with tumor tissue flaps in the right or left dorsal area of the trunk. The skin was cleaned using 70% ethanol and a small skin incision was made. The tumor tissue flap was then introduced into the subcutaneous pocket with a transplantation needle (ϕ 2.0 mm). After injection, the tumor sizes were measured once a week. Putative tumor volume (V) was calculated as (width)^2 ^× length/2 mm^3^. Tumor volumes were then expressed as fractional tumor volumes (V/V_0_), where V_0 _is the initial volume of the tumor immediately before treatment (day 0). When the tumors had increased to approximately 10 mm in diameter, tumors growing in nude mice were serially transplanted from mouse to mouse. The mice were scarified, and the tumor was immediately removed. Subsequently, several pieces of the xenografted tumor tissues were stored in a freeze-stock solution (Cell Banker; Nippon Zenyaku Kogyo Co., Fukushima, Japan) in a deep freezer at -80°C to confirm the tumorigenicity of frozen xenografted tumor tissues. All the excised tumors were treated the same as the spontaneous tumors for histopathological analyses. Snap-frozen samples were also collected for reverse transcriptase polymerase chain reaction (RT-PCR) and stored at -80°C until use. The experiments were performed according to the guidelines for the care and use of laboratory animals approved by the Animal Care and Use Committee of Gifu University.

### Immunohistochemical Staining of Original or Xenograft Tumors

Immunohistochemical staining was performed for CD31 (anti-human CD31 mouse monoclonal antibody, prediluted; Dako Cytomation, Glostrup, Denmark), von Willebrand factor (anti-human von Willebrand factor rabbit monoclonal antibody [vWF], prediluted; Dako Cytomation), Ki-67 antigen (anti-human Ki-67 antigen mouse monoclonal antibody, clone MIB-1, 1:25; Dako Cytomation), VEGF-A (anti-human VEGF [C-1] mouse monoclonal antibody, 1:50; Santa Cruz Biotechnology, Santa Cruz, CA, USA), flt-1 (anti-human flt-1 [C-17] rabbit polyclonal antibody, 1:200; Santa Cruz Biotechnology), flk-1 (anti-mouse flk-1 [A-3] mouse monoclonal antibody, 1:200; Santa Cruz Biotechnology), bFGF (anti-human FGF-2 [147] rabbit polyclonal antibody, 1:200; Santa Cruz Biotechnology), HoxA9 (anti-human HoxA9 [A-20] goat polyclonal antibody, 1:200; Santa Cruz Biotechnology), HoxB3 (anti-human HoxB3 [C-20] goat polyclonal antibody, 1:100; Santa Cruz Biotechnology), HoxB7 (anti-human HoxB7 rabbit polyclonal antibody, 1:1,000; CeMines, Golden, CO, USA), HoxD3 (anti-human HoxD3 rabbit polyclonal antibody, 1:400; Aviva Systems Biology, San Diego, CA, USA), Pbx1 (anti-human Pbx1 rabbit polyclonal antibody, 1:100; Novus Biologicals, Littleton, CO, USA), and Meis1 (anti-human Meis1 rabbit polyclonal antibody, 1:200; Abcam, Tokyo, Japan) for all the original tumor and xenograft samples that had been transplanted more than 5 times consecutively and then transplanted into frozen xenografted tumor tissues. These antibodies have been reported to be able to react with peptides of canine origin [10,24-27]. Antibodies to HoxB7, Pbx1, and Meis1 are purified against a synthetic peptide whose sequence is identical to the canine peptide [28]. Although the antibodies to HoxA9, HoxB3, and HoxD3 are purified against corresponding peptides of the human protein regions, it is unclear whether or not these sequences corresponded to protein sequences in canines. To demonstrate the staining characteristics for those antibodies in angiogenic status, canine granulation tissue was immunohistochemically stained. HoxD3 was used at a dilution attained by adding Can Get Signal.^® ^immunostain Solution A (Toyobo, Osaka, Japan). Ki-67 antigen, VEGF-A, flt-1, flk-1, bFGF, HoxA9, HoxB3, HoxB7, Pbx1, and Meis1 were diluted by adding 1% bovine albumin/phosphate-buffered saline (PBS). In brief, 3-μm sections were deparaffinized in Lemosol (Wako Pure Chemical Industries, Osaka, Japan) and rehydrated in graded ethanol. For the antigen retrieval of vWF, flk-1, flt-1, HoxA9, HoxB3, HoxB7, Pbx1, and Meis1, sections were immersed in Target Retrieval Solution (Dako Cytomation) and heated for 15 min at 121°C in an autoclave. For antigen retrieval of Ki-67, sections were immersed in Target Retrieval Solution and heated for 25 min at 121°C in an autoclave. For demasking CD31, sections were digested with 0.5% proteinase K (Takara, Ohtsu, Japan) in Tris-HCl buffer (pH 7.0) for 5 min at 37°C. For demasking VEGF-A and bFGF, sections were digested with 0.1% trypsin (GIBCO Life Technologies, NY, USA) in Tris-HCl buffer (pH 7.0) for 15 min at 37°C. Endogenous peroxidase was blocked by incubation in 0.3% H_2_O_2 _in methanol for 20 min at room temperature (RT). To prevent the binding of nonspecific proteins to the primary antibody, the sections were treated with Protein Block Serum-Free (Dako Cytomation) for 30 min at RT. Sections were incubated with the primary antibody overnight at 4°C; for the negative control, PBS was used in place of the primary antibody. The sections were then washed with PBS and incubated with the appropriate secondary antibodies (Histofine.^® ^simple stain MAX PO; Nichirei, Tokyo, Japan) at RT for 30 min. After washing thrice with PBS, binding was detected using 3,3'-diaminobenzidinetetrahydrochloride (Liquid DAB + Substrate Chromogen System; Dako Cytomation). Finally, the sections were washed in distilled water and counterstained with Mayer's hematoxylin.

The percentage of Ki-67-positive cells in each tumor tissue was calculated as the number of reddish brown-positive nuclei in a total of 1000 neoplastic cells (from 10 high-power fields); this value is indicated as a Ki-67 positive index (PI).

### Detection of mRNA Expression by RT-PCR

Total RNA was extracted from 6 xenograft tumors using TRIzol reagent (Gibco Life Technologies). In brief, a snap-frozen sample was homogenized in 1 ml TRIzol by a polytron-type homogenizer and mixed with 0.2 ml chloroform before centrifugation. The aqueous phase was collected for total RNA extraction, mixed with 2-propanol to salt out, and washed with chilled 75% ethanol. Total RNA was detected after treatment with amplification-grade DNase I (Invitrogen, Carlsbad, CA, USA) followed by RT-PCR assay with Qiagen One step RT-PCR Kit (Qiagen, Valencia, CA, USA), according to the manufacturer's instructions. RT-PCR was carried out in a Thermal Cycler Dice Gradient (Takara). Amplifications were performed under the following conditions: reverse-transcription reaction for 30 min at 50°C, an initial polymerase activation step for 15 min at 95°C, denaturation for 30 s at 95°C, annealing for 30 s, extension for 1 min at 72°C, and a final extension for 10 min at 72°C. To confirm the omission of genomic contamination, RT-PCR was carried out for DNase I-treated total RNA with One Step Enzyme Mix, which was deactivated for reverse transcription activity by heating at 95°C for 15 min.

The primer sequences, annealing temperatures, annealing cycle number, product sizes used, and the accession number for generating the primer sets are listed in Additional file 1. Each primer used in this study was generated from canine-specific sequences, by accession number (e.g., Genbank). The sequences that the primers recognize in the sandwich splicing area and the predicted sizes differed among VEGF variants. The primers amplifying HoxA9 mRNA distinguished between HoxA9 mRNA, which contains the region encoding the homeodomain, and the HoxA9 mRNA lacking this region. RNA integrity was confirmed by the sufficient amplification of β-actin. The specificity of all canine primer sets was definitive, because there was no band in the RT-PCR for normal mouse spleens. The products of the PCR amplifications were electrophoresed in 2% agarose gel or 7.5% polyacrylamide gel for VEGF stained with ethidium bromide and visualized under ultraviolet light.

## Results

### Tumor Xenografts

**Figure 1:**
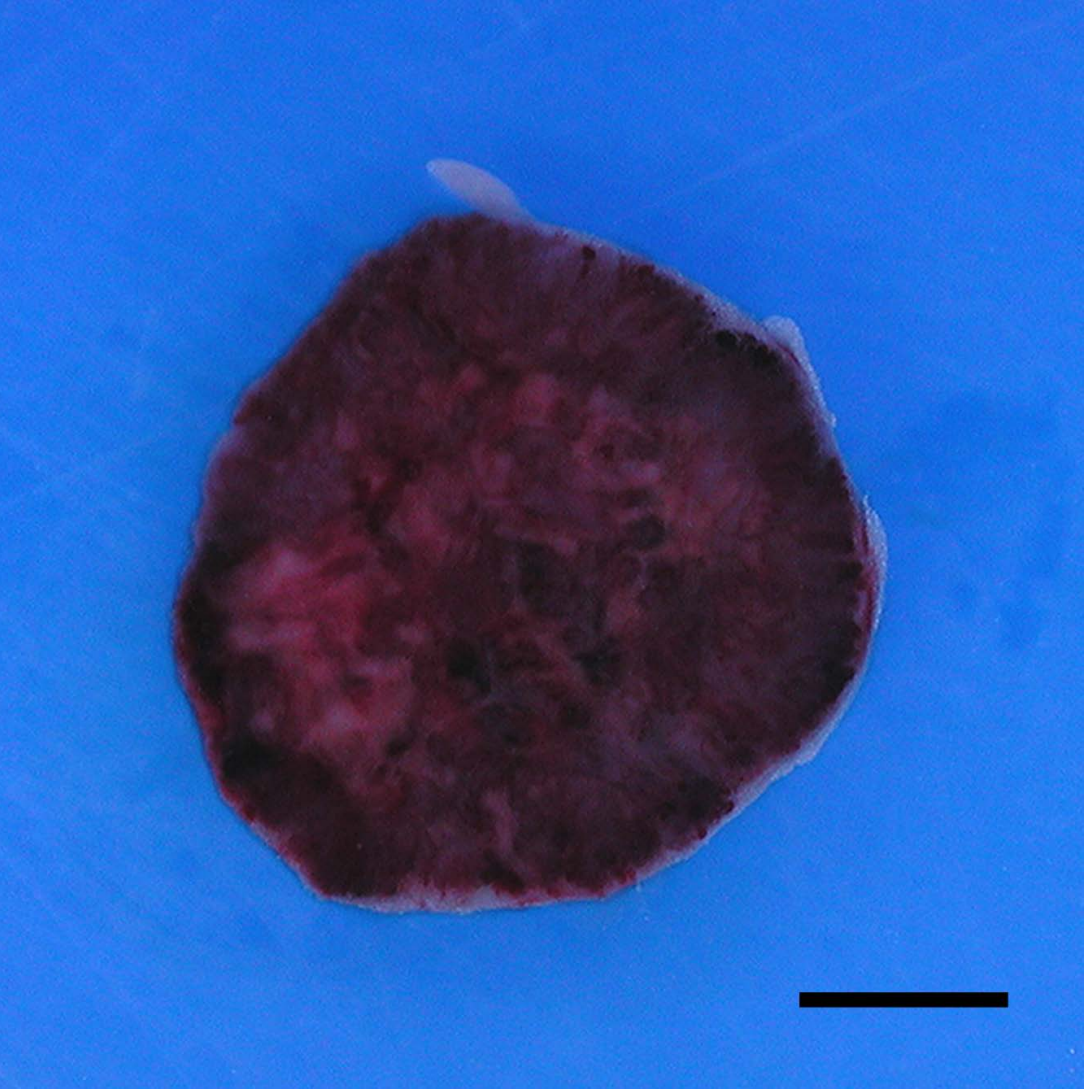
**Gross appearance of Ud xenograft tumors at 24 days after transplantation, which was transplanted repeatedly 20 times**. The xenograft tumor was solid, dark red in color, and contained necrotic and blood-filled areas on the cut surface. Bar: 2.5 mm.

Of the 13 spontaneous canine HSA xenografts we attempted to establish in nude mice, 6 were successful and resulted in 6 growing canine HSA tumors. All these tumors were serially transplanted from mouse to mouse at least 5 times and had been maintained for over a year on nude mice. All the tumors grew to large masses (approximately 500 mm^3 ^each) in the subcutis of nude mice, at 3-6 weeks after the pieces of the original tumors had been inoculated (Figure 1). There were no significant changes in the growth rate through passages in all xenograft tumors (data not shown). All frozen samples from xenograft tissues successfully formed tumors in nude mice. With the exception of tumor formation, no clinical abnormalities were observed in any of the transplanted mice during their experimental term. Grossly, none of the xenograft tumors adhered to underlying muscles or overlying skins, and the tumors were discretely formed. The tumors were soft and had a dark reddish hue on the cut surface. At necropsy, no metastatic lesions were observed in any of the organs.

### Histopathology

**Figure 2:**
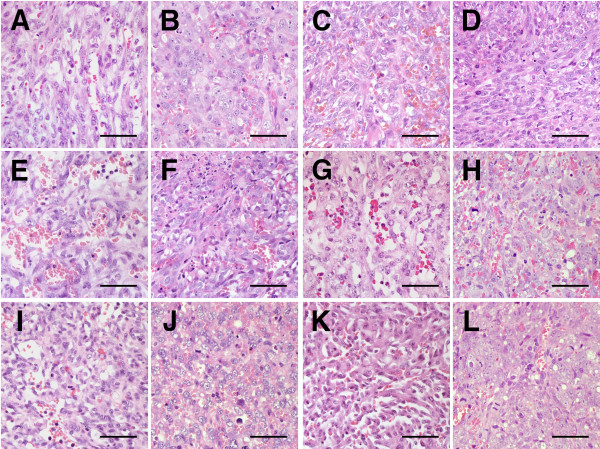
**Histological features of original HSAs and xenograft HSA tumors**. Microscopic features of original HSAs and xenograft HSA tumors. Histologically, each of the original HSAs (A, C, E, G, I, and K present the original tumors of Si, Re, Ud, Sy, Sa, and Ju, respectively) showed vascular-like proliferation and formed solid sheets with various-sized clefts. The neoplastic cells that comprised original tumors had various shapes, ranging from spindle-shaped and polygonal to ovoid. Each of the xenograft tumors (B, D, F, H, J, and L present Si, Re, Ud, Sy, Sa, and Ju, respectively) were predominantly solid and contained poorly formed, irregular-shaped vascular spaces with erythrocytes, neutrophils, and lymphocytes. In all xenograft tumors, the neoplastic cells became more pleomorphic, elongated, and plump and the nuclei became larger and more clearly polygonal in shape. Hematoxylin and eosin (HE); bars: 50 μm.

Histopathologically, all original tumors comprised proliferations of neoplastic cells that were highly variable in shape, ranging from spindle-shaped and polygonal to ovoid (Figure 2A, C, E, G, I, and 2K). There were some vascular-like structures and vascular clefts of channels that contained erythrocytes, neutrophils, and lymphocytes. Similarly, all xenograft tumors, including the tumors from the frozen xenografts, comprised clusters of small sheets of pleomorphic to plump spindle cells that frequently formed slit-like spaces, many of which also contained erythrocytes, neutrophils, and lymphocytes (Figure 2B, D, F, H, J, and 2L). The nuclei of neoplastic cells comprising the xenograft tumors sustained more severe anisokaryosis than those in the original tumors. Necrotic foci were scattered. Similar to original tumors, mitotic figures were frequent in all xenograft tumors. The neoplastic cells invaded into the subcutaneous adipose tissue in the peripheral area of neoplastic proliferation. Compared to the original tumors, the xenograft tissues tended not to form vascular-like clefts of channels.

### Immunohistochemical Analysis

**Figure 3:**
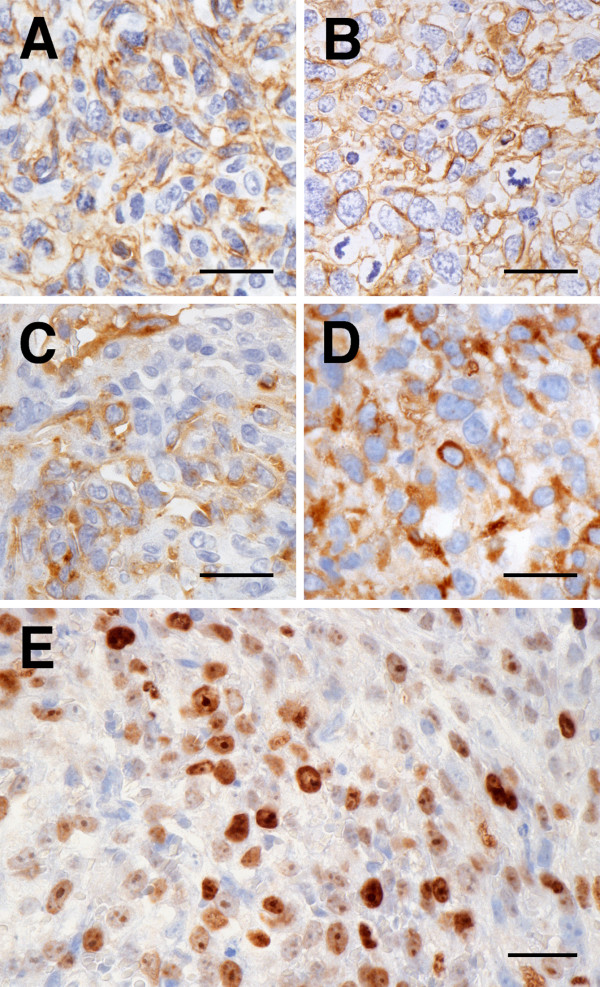
**Endothelial cell-specific markers and Ki-67 immunolabeled in original HSAs and xenograft HSA tumors**. Immunolabeling of CD31, vWF, and Ki-67, in original HSAs and xenograft HSA tumors. All original HSAs had membrane immunoreactivity for CD31 (A: the original tumor of Sa) and cytoplasmic immunoreactivity for vWF (C: the original tumor of Sa). Immunoreactivity for CD31 (B: Sa) and vWF (D: Sa) in all xenograft HSA tumors was detected in the same location as in the original tumors. In all xenograft HSA tumors, Ki-67 (E: Sy) nuclear immunoreactivity was observed in the nuclei of the tumor cells that were derived from canine HSA. Immunohistochemistry (IHC); bars: 50 μm.

**Figure 4:**
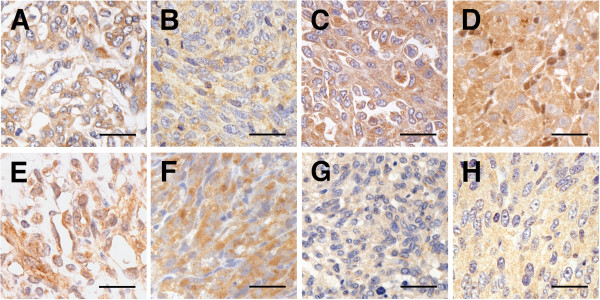
**Angiogenic growth factors and their receptors immunolabeled in original HSAs and xenograft HSA tumors**. Representative immunohistochemical results of VEGF-A, flt-1, flk-1, and bFGF in original HSAs and xenograft HSA tumors. All original HSAs had cytoplasmic immunoreactivity for VEGF-A (A: the original tumor of Re), flt-1 (C: the original tumor of Ju), flk-1 (E: the original tumor of Ud), and bFGF (G: the original tumor of Si). Immunoreactivity for VEGF-A (B: Re), flt-1 (D: Ju), flk-1 (F: Ud), and bFGF (H: Si) in all xenograft HSA tumors was detected in the same location as in the original tumors. Immunohistochemistry (IHC); bars: 50 μm.

**Figure 5:**
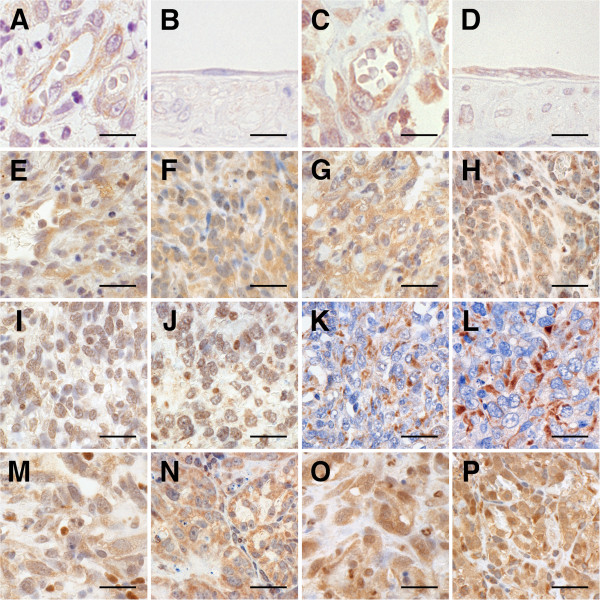
**Angiogenesis-associated homeobox proteins immunolabeled in canine normal ECs, original HSAs, and xenograft HSA tumors**. Representative immunohistochemical results of HoxD3 and Pbx1 in active and quiescent ECs during angiogenesis. In granulation tissue, although immunoreactivity for HoxD3 (A) and Pbx1 (C) was localized in the nuclei and cytoplasm of angiogenic ECs, it was weak for HoxD3 in the nuclei. Immunoreactivity for HoxD3 (B) was not detected in quiescent ECs but for Pbx1 (D), the immunoreactivity was localized in the nuclei and cytoplasm of quiescent ECs. Representative immunohistochemical results of HoxA9, HoxB3, HoxB7, HoxD3, Pbx1, and Meis1 in original HSAs and xenograft HSA tumors. In all original HSAs, immunoreactivity for HoxA9 (E: the original tumor of Ud), HoxB3 (G: the original tumor of Sy), HoxD3 (K: the original tumor of Sa), Pbx1 (M: the original tumor of Ud), and Meis1 (O: the original tumor of Ud) was localized in the nuclei and cytoplasm of the neoplastic cells. HoxB7 (I: the original tumor of Sa) immunoreactivity was only localized in the nuclei of the neoplastic cells. Immunoreactivity for HoxA9 (F: Ud), HoxB3 (H: Sy), HoxB7 (J: Sa), HoxD3 (L: Sa), Pbx1 (N: Ud), and Meis1 (P: Ud) in all xenograft HSA tumors was detected in the same location as those original tumors. IHC; bars: 25 μm (A--D) and 50 μm (E--P).

The presence of CD31 and vWF--both of which confirmed the endothelial nature of the tumors--was detected by immunohistochemical staining in all original tumors and xenograft tumors including the tumors from frozen xenografts (Figure 3A-D). CD31 was positive for the cytoplasmic borders of neoplastic cells and vWF was positive for diffuse or patchy cytoplasm. The neoplastic cells were also positive for MIB-1 (Figure 3E). The Ki-67 PIs of the xenograft samples (mean PI, 55.7%; range, 47.2-70.7%) were equal to or higher than those of the original tumors (mean PI, 39.3%; range, 28.6-51.2%) in all tumors. Immunohistochemical staining on all xenograft tumors, including the tumors from frozen xenografts, and original tumors were positive for antibodies against VEGF-A, flt-1, flk-1, and bFGF (Figure 4). Most ECs in the proliferating capillaries of canine granulation tissue were labeled by primary antibodies for HoxA9, HoxB3, HoxB7, HoxD3, Pbx1, and Meis1, and the proteins were expressed in both the nucleus and the cytoplasm. HoxD3 (Figure 5A) and Pbx1 (Figure 5C) had weaker expression in the nucleus than in the cytoplasm of these cells. In contrast, quiescent ECs in mature vessels had strong nuclear expression of HoxB3 and Meis1, but their expression in the cytoplasm was weak or absent. HoxA9 and HoxB7 were positive only in the nuclei of these cells, and HoxD3 (Figure 5B) and Pbx1 (Figure 5D) were absent or weakly positive in quiescent ECs. HoxA9, HoxB3, Pbx1, and Meis1 were strongly expressed within the nuclei and cytoplasm of neutrophils and macrophages, but HoxB7 expression was restricted to the nuclei of these cells. Moreover, HoxA9, HoxB3, HoxB7, HoxD3, Pbx1, and Meis1 were positive in all the original tumors and xenograft tumors, including the tumors from frozen xenografts (Figure 5E-P). HoxA9, HoxB3, HoxD3, Pbx1, and Meis1 were positive in the nuclei and cytoplasm of neoplastic cells in all original tumors and xenograft tumors. Cytoplasmic immunoreactivity for HoxA9, HoxB3, Pbx1, and Meis1 was found to be diffuse in all original and xenograft tumors, and cytoplasmic immunoreactivity for HoxD3 was also found to be diffuse or localized in all original tumors and xenograft tumors, particularly in the perinuclear area. HoxB7 was strongly positive for the nuclei of neoplastic cells in all original tumors and xenograft tumors.

### Expression of CD31, vWF, VEGF-A, flt-1, flk-1, bFGF, FGFR-1, EphB4, and Angiogenic Homeobox Genes

**Figure 6:**
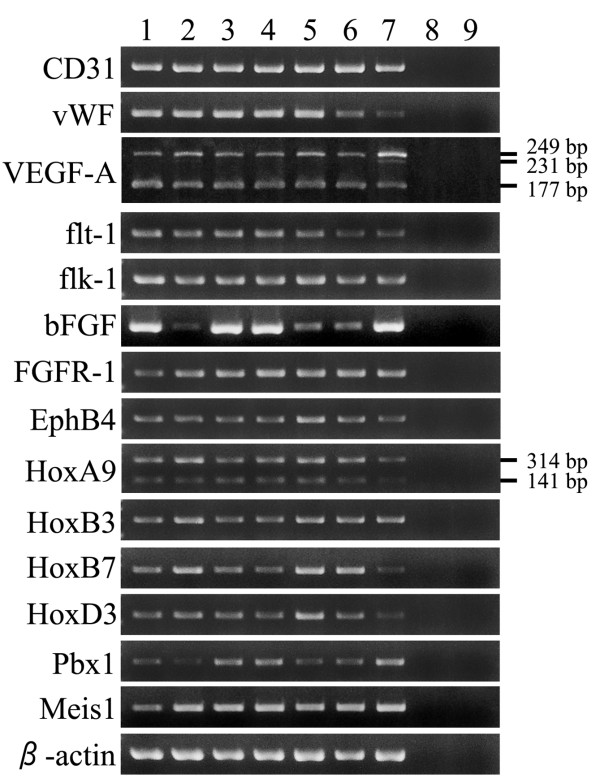
**Gene expressions of neoplastic cells in xenograft HSA tumors**. RT-PCR analyses of the expression of endothelial cell-specific markers (CD31 and vWF), angiogenic growth factors/receptors (VEGF-A, flt-1, flk-1, bFGF, and FGFR1), and homeobox genes (HoxA9, HoxB3, HoxB7, HoxD3, Pbx1, and Meis1) in the xenograft HSA tumors (1: Si, 2: Re, 3: Ud, 4: Sy, 5: Sa, 6: Ju, 7: normal canine spleen, 8: normal mouse spleen, and 9: negative control of RT-PCR). The mRNAs of endothelial cell-specific markers, angiogenic growth factors/receptors, and homeobox genes were detected in all the xenograft HSA tumors. In particular, the mRNAs of VEGF-A_164 _(177-bp) and VEGF-A_188 _(249-bp) were detected in all the xenograft HSA tumors, but VEGF-A_182 _(231-bp) was not present. The 2 variants of HoxA9 mRNA (314-bp and 141-bp bands) were detected in all the xenograft HSA tumors.

The results of RT-PCR are shown in Figure 6. In the amplification of VEGF-A mRNA by RT-PCR, 2 major bands (177- and 249-bp bands corresponding to VEGF-A_164 _and VEGF-A_188 _mRNA, respectively) were detected in all xenograft tumors and normal canine spleen samples; however, the 231-bp band corresponding to VEGF-A_182 _was not present. Amplification for CD31, vWF, flt-1, flk-1, bFGF, FGFR-1, and EphB4 mRNA yielded single 211-, 221-, 156-, 187-, 234-, 162-, and 178-bp products, respectively, in all xenograft tumors and normal spleen samples. The expression of bFGF mRNA was more obvious in the Si, Ud, and Sy xenograft tumors. In the amplification of the HoxA9 mRNA by RT-PCR, 2 bands (314-bp and 141-bp bands corresponding to HoxA9 mRNA containing the region encoding the homeodomain and HoxA9 mRNA lacking it, respectively) were detected in all xenograft tumors and normal canine spleen samples. The 141-bp band was less intense than the 314-bp band in all the xenograft tumors and normal canine spleen samples. Amplification for HoxB3, HoxB7, HoxD3, Pbx1, and Meis1 mRNA yielded single 198-bp, 243-bp, 165-bp, 232-bp, and 284-bp products, respectively, in all xenograft tumors and normal spleen samples.

## Discussion

In this study, 6 xenograft canine HSAs were established. As compared to the original tumors, xenograft tissues tended to form neither vascular-like clefts of channels nor higher nuclear atypia of xenograft tumors; however, the histopathological structures of xenograft tumors were similar to those in original canine HSA, and mRNA expressions and immunoreactivities of vWF and CD31 were also detected in the xenograft tumors. Thus, the xenograft tumors in the present study maintained the canine endothelial nature. In immunohistochemistry for Ki-67 antigen using antibody clone MIB-1, xenograft tumor cells were clearly positive in the nucleus but negative in the surrounding murine tissues such as the epidermal basal cells. The MIB-1 antibody did not react with murine homologous antigen but reacted with canine homologous antigen; thus, xenograft tumors were confirmed as being of canine origin [29]. Some xenograft tumors indicated higher Ki-67 PI values than those of original tumors. Thus, given the atypia and the Ki-67 PI of the original and xenograft tumors, it is suggested that more malignant components may be selected by repeated xenotransplantation.

In spontaneous canine HSAs, the mRNA of VEGF-A and bFGF and their receptors were detected; however, they can be immunohistochemically detected not only in neoplastic cells but also in inflammatory cells [10]. Actually, the results of RT-PCR in spontaneous canine HSA uncovered the presence of both neoplastic cells and infiltrating inflammatory cells [10]. Thus, contaminated inflammatory cells could disrupt HSA analysis, especially the detection of gene expression; it is difficult to resolve this problem when using solely clinical samples. We attempted to establish xenograft models in the present study, as they are powerful tools in resolving this problem. Because the expression of VEGF-A, flt-1, flk-1, bFGF, and FGFR-1 mRNA was detected in xenograft tumors by the canine-specific primers, amplified products originated precisely from canine neoplastic ECs. These results suggest that accurate gene expression could be detected in the xenograft models established in the present study. In addition, xenograft HSA tumors showed positive immunolabeling for VEGF-A, bFGF, flt-1, and flk-1, similar to the original tumors; thus, those xenograft models maintain a proliferative mechanism similar to that found in spontaneous canine HSA.

There is increasing evidence that the dysregulation of molecular pathways governing angiogenesis may be important to HSA pathogenesis [30]. Yonemaru *et al*. suggest that canine HSA might have an autocrine or a paracrine mechanism via VEGF-A and/or bFGF receptor(s) [10]. VEGF-A, a strong angiogenic factor, is expressed at low levels in normal mammalian tissues; increased levels of VEGF-A were observed during ischemia or hypoxia, and in lesions characterized by angiogenesis. In a recent study [31], VEGF-A_120_, VEGF-A_164_, and VEGF-A_188 _were detected in normal canine tissues, and VEGF-A_164_, which corresponds to human VEGF-A_165_, is the dominant molecular species. Canine VEGF-A_164 _contains all the necessary information for the normal growth, remodeling, and patterning of blood vessels [32]. Some reports investigate the expression of VEGF-A variants in various human neoplasms [33-39]. The increased expression of human VEGF-A_165 _and VEGF-A_189 _in osteosarcoma [33], renal cell carcinoma [34], non-small-cell lung cancer [35-37], and colon cancer [38] correlates with neovascularization, tumor progression, and poor prognosis. In human angiosarcoma, the expression of VEGF-A mRNA has been demonstrated, but the expression of VEGF-A variants has not been investigated [40]. VEGF-A_120 _and VEGF-A_164 _have been detected as major bands in canine HSA, while VEGF-A_144 _and VEGF-A_188 _have been detected as minor bands; however, spontaneous HSA contains contaminating inflammatory cells that express VEGF-A mRNA [10]. In the present study, primers for VEGF-A were expected to produce 3 splicing variants, namely, VEGF-A_164_, VEGF-A_182_, and VEGF-A_188_. VEGF-A_164 _and VEGF-A_188 _but not VEGF-A_182 _were detected in all xenograft tumors and normal canine spleens. VEGF-A_164 _has been demonstrated to have a higher expression as compared to the expression of VEGF-A_182 _and VEGF-A_188 _in both spontaneous and xenograft HSA tumors.

The expression of bFGF mRNA was different among the xenografts. In 3 of the xenografts--Si, Ud, and Sy--the expression levels of bFGF mRNA were found, by RT-PCR, to be higher than those of other xenografts. The intensity of specific bands for bFGF was similar to those of normal canine spleen samples, which were used as a positive control; however, spleens contain various cells--including leukocytes, ECs, and other stromal cells--and thus the actual expression of bFGF in splenic ECs might be low. Therefore, the expression levels of bFGF mRNA in Si, Ud, and Sy might be high and must be measured quantitatively. bFGF is one of the most effective angiogenic growth factors; therefore, it might play a role in malignant endothelial growth in HSA.

In recent studies, it has been demonstrated that several homeobox genes regulate the behavior of ECs during angiogenesis and it is suggested that the homeobox proteins of HoxA9, HoxD3, and Pbx1 are involved in the malignancy of canine HSA [13,17,27]. HoxA9, HoxB3, HoxB7, and HoxD3 proteins regulate the expression of downstream genes involved in angiogenesis; Pbx1 and Meis1 assist in the functioning of Hox protein, as cofactors [16,41]. In the present study, immunostaining of xenograft HSA tumors was positive for HoxA9, HoxB3, HoxB7, HoxD3, Pbx1, and Meis1, which is similar to the proteins detected by immunostaining of the original tumors. The expression of HoxA9, HoxB3, HoxB7, HoxD3, Pbx1, and Meis1 mRNA was also detected in all xenograft tumors. HoxA9 mediates EC migration and proliferation during angiogenesis by regulating the expression of EphB4, a member of the Eph receptor tyrosine kinase family [13,42]. HoxA9 binds the EphB4 promoter and induces the transcription of the EphB4 mRNA directory [13]. Therefore, the expression of EphB4 mRNA in all xenograft tumors suggests that HoxA9 may induce the expression of EphB4 in HSA. Moreover, HoxA9 has been found to be a key regulator that induces acute myeloid leukemia in mice [43]. Although coactivation of HoxA9 and Meis1 in mouse bone marrow cells has reportedly induced acute myeloid leukemia rapidly, this phenomenon was not observed in terms of an individual overexpression of each of the homeobox genes [41]. The function of HoxA9 in HSA may have been enhanced by the presence of Meis1.

HoxD3 both induces an angiogenic phenotype and enhances the expression of both integrin αvβ3 and urokinase-type plasminogen activator--the latter of which, in turn, facilitates EC migration and adhesion during bFGF-induced angiogenesis [17,44]. In human hemangioma, expression levels of HoxD3 mRNA in the proliferation phase are higher than those in the involution phase [45]. Pbx1 is known to be necessary for angiogenesis mediated by HoxD3 [16]; therefore, one of the functions of HoxD3 is to induce ECs so that they are in an active phase--proliferative and migratory--and it is suggested that HoxD3 is involved in the tumorigenesis of canine HSA. Moreover, HoxB3 promotes capillary morphogenesis during angiogenesis, and HoxB7 directly upregulates the expression of bFGF in human melanoma cells; the activation of bFGF induces EC proliferation *in vitro *[14,46]. Finally, in 2 recent studies, it was demonstrated that Pbx1 functions as a cofactor with HoxB3 and HoxB7 [47,48]. The result--that HoxB7 and bFGF are expressed in HSA--may suggest that HoxB7 induces the proliferation of tumor cells mediated by bFGF.

The cytoplasmic localization of HoxA9, HoxB3, HoxD3, Pbx1, and Meis1 proteins in original tumors was maintained in all xenograft HSA tumors. Previous studies have shown the cytoplasmic localization of homeobox proteins, suggesting modifications in their localization during tumorigenesis [49-51]. The localization of homeobox proteins might be involved in tumorigenesis in HSA. Although xenograft models that demonstrate the expression of homeobox genes have been reported in human small-cell lung cancers, there is no model available to analyze homeobox expression in HSA [52]. Recent studies have indicated the possibility of a novel tumor therapy that involves regulating aberrant expressions of homeobox genes [53-55]; Plowright *et al*., for example, suggest that the interaction between Hox and Pbx proteins is a potential therapeutic target [56]. Therefore, the present xenograft HSA models may be useful in evaluating a novel molecularly targeted therapy involving homeobox protein.

In this study, no metastatic lesion was observed in any of the xenograft tumors. Although there might not have been sufficient time for the tumor to metastasize, the occurrence of metastasis depends on the mouse strains in the xenograft tumors and tumor cell lines [57-59]. Therefore, the metastasis model of xenografted canine HSAs may be established by the transplanting the present xenograft tumors into SCID or NOD-SCID mice. In general, orthotopic transplanted tumors have a greater tendency than heterotopic transplanted tumors to metastasize to any organ [60-62]. Therefore, orthotopic canine HSA transplantation may lead to the development of metastatic lesions in nude mice. It may be possible that the xenograft tumors lost the expression of the factors required to develop metastasis. Alternatively, metastasis-promoting factors produced by xenograft canine tumors might not react with mice. However, the relationship between metastasis and the factors could not be elucidated in this study.

Since the chemotherapy and radiotherapy was not carried out in this study, the effects of these therapies for the xenografted models are unclear. In medicine, surgical resection and radiotherapy for HSA has been reported to result in long-term survival in many cases [3,4]. On the other hand, no standard chemotherapy for HSA has been established; nevertheless, several approaches in chemotherapy have been attempted, such as liposomal doxorubicin, interleukin-2, and interferon-α [63-65]. In recent years, anti-angiogenic therapy has attracted considerable attention as a possible therapy for HSA [66-68]. Given the inferred mechanism of proliferation via VEGF-A or bFGF in HSA, anti-angiogenic therapy would be the logical strategy for the treatment of HSA [9,10]. Therefore, it is hoped that the xenografted models, which are HSA tumors producing VEGF-A, bFGF, and their receptors, will be useful to evaluate the efficacy of the novel drug that inhibits VEGF- or bFGF-mediated neoplastic proliferation in HSA.

## Conclusion

In the present study, 6 murine xenograft canine HSA tumors were established. All xenograft tumors expressed CD31, vWF, VEGF-A, bFGF, and their receptors; thus, these tissues maintained endothelial characteristics, especially active ECs. Moreover, angiogenic homeobox genes were expressed in the xenograft tissues. Together, these xenograft canine HSA tumors constitute the first model to demonstrate the expression of homeobox genes in HSA and canine tumors. Since the tumors from the frozen xenografts replicated the characteristics of original tumors, the models are capable of reproducing xenografted HSA tumors at any time. They may contribute to elucidating the mechanism of HSA proliferation and aid in the development of novel HSA therapy for dogs and humans alike.

## Competing interests

The authors declare that they have no competing interests.

## Authors' contributions

AK participated in the design of the study; dissected the animals used; performed histopathology, immunohistochemistry, and molecular genetic studies; and drafted the manuscript. HS conceived the aims of study, and participated in its design and coordination. SM, MM, and AM dissected the animals and carried out immunohistochemical analyses. TM and KM prepared the spontaneous canine HSAs for transplantation. TK carried out the dissection of the animals and the molecular genetic studies. TM and TY initiated the study and participated in its coordination. All the authors have read and have approved of the final manuscript.

## Pre-publication history

The pre-publication history for this paper can be accessed here:

http://www.biomedcentral.com/1471-2407/9/363/prepub

## Supplementary Material

Additional file 1 **PCR primers and conditions**. The data provided represent the sequences of canine-specific primer sets and the PCR conditions for those primer sets used in the present study. Click here for file
